# Effect of *Streptococcus anginosus* on biological response of tongue squamous cell carcinoma cells

**DOI:** 10.1186/s12903-021-01505-3

**Published:** 2021-03-20

**Authors:** Yuan Xu, Yuhuan Jia, Liang Chen, Jing Gao, DeQin Yang

**Affiliations:** 1 College of Stomatology, Chongqing Medical University, Songshi Road No. 426, Yubei District, Chongqing, China; 2 Chongqing Key Laboratory of Oral Diseases and Biomedical Sciences, Songshi Road No. 426, Yubei District, Chongqing, China; 3 Chongqing Municipal Key Laboratory of Oral Biomedical Engineering of Higher Education, Songshi Road No. 426, Yubei District, Chongqing, China

**Keywords:** *Streptococcus anginosus*, Oral squamous cell carcinoma, Autophagy, Proliferation, Apoptosis

## Abstract

**Background:**

*Streptococcus anginosus* (*S. anginosus*) was reported increased in oral squamous cell carcinoma (OSCC) tissue. The aim of this study was to investigate the response of oral cancer cells in the biological characteristics evoked by the *S. anginosus and investigate its potential mechanisms.*

**Methods:**

The growth curve and concentration standard curve of *S. anginosus* were determined, and a series of concentrations of *S. anginosus* supernatant were applied to OSCC cell lines SCC15, then selected an optimal time and concentration by CCK-8 assay. Then autophagic response, proliferative activity, cell cycle and apoptosis, invasion and migration abilities were evaluated in SCC15.

**Results:**

The results showed that when the ratio of *S. anginosus* supernatant to cell culture medium was 1:1 and the co-culture time was 16 h, the inhibitory effect on SCC15 was the most obvious; Furthermore, the supernatant of *Streptococcus* upregulated the autophagy activity of SCC15, thus significantly inhibiting its proliferation, migration and invasion ability. Compared with control groups, the cell cycle showed G1 arrest, S and G2/M phases decreased, and the percentage of apoptotic cells relatively increased (*P* < 0.05).

**Conclusion:**

*S. anginosus* reduced the proliferation, migration and invasion of SCC15 cells and promoted cell apoptosis; Moreover, autophagy may be one of the mechanisms in this process.

**Supplementary information:**

The online version contains supplementary material available at 10.1186/s12903-021-01505-3.

## Background

In the 1990s, the researchers found that there may be a relationship existed between microbes and tumors [1]. Studies have showed that the primary cause of carcinogenesis was the changes in cellular metabolism [2]. Microorganisms participate in a series of metabolic activities of the host, and the metabolites or co-metabolites (produced by the interaction between the host and the microorganisms) of microorganisms will affect the proliferation and apoptosis of tissue cells and the growth and metastasis of cells in the tumor microenvironment [3, 4]. Currently, the application status of microorganisms in cancer treatment includes: as a natural medicine database, bacteria provide various active substances with anti-tumor properties; As a delivery vehicle, bacteria specifically target drugs to tumor cells; As a therapeutic agent, bacteria combining with chemoradiotherapy and immunotherapy synergistically promote the efficacy of traditional anti-tumor therapy [5].

The human digestive flora is the second set of human genomes, which inhabits a large number of microorganisms, and the number of which is 10 times the number of their own cells. The micro-ecological system composed of these bacteria maintains the stability and balance of the microbial community under their interaction. In recent years, with the development of technologies like metagenomics and high-throughput sequencing, research on microbes and human health and diseases has gradually started. More and more studies have found that in some digestive tract tumors, the composition of the flora has changed significantly, and some bacteria have increased or decreased in specificity. Helicobacter pylori has been identified as an independent risk factor for gastric cancer, while studies have also reported that beneficial bacteria such as *bifidobacteria* can inhibit the occurrence and development in gastric tumors. Therefore, scholars have proposed that microbes could be regarded as a double-edged sword in tumors. The metabolism of cancer cells will become a promising therapeutic target, and the role of microorganisms in cancer treatment is becoming more and more important [6].

The oral cavity is not only the initiation site of digestive tract but also the predilection site of head and neck malignant tumors, and 90% of oral cancers are squamous cell carcinoma. Studies have found that patients with oral squamous cell carcinoma (OSCC) have some significant changes in the microbial diversity of cancer tissues and saliva compared with healthy people, such as *Streptococcus, Porphyromonas, fusobacterium, Veillonella, Actinomyces, Enterobacter* and so on [7–9]. *S. anginosus* is a commensal bacterium in the oral cavity, gastrointestinal tract and genitourinary tract. Previous studies have shown that the detection rate of this strain in esophageal cancer and oral squamous cell carcinoma is significantly increased and the pathogenicity in esophageal cancer has been clear [10–12]. The oral cavity connects with esophagus, some researchers considered that this kind of bacterium may involve in oral squamous cell carcinoma [13, 14]. A previous study on the cancer tissues of different sites in oral cavity estimated that the total detection rate of *S. anginosus* DNA was as high as 88%, and each part was 87% of tongue, 90% of mouth, 83% of cheek, 100% of gums, and this rate was found much less frequently in healthy people [15].

In this study, we estimated the changes of SCC15 cells fate by *S. anginosus* and explored the potential mechanism that reveal the impact of this bacterium on the biological characteristics of oral cancer cells.

## Methods

### Baterial growth curve and concentration standard curve

*S. anginosus* strain ATCC33397 (supported by the State Key Laboratory of Oral Disease, Sichuan University) was cultured aerobically at 37 °C in THB medium (Solarbio, China) and measure the absorbance every 2 h. Then the relationship between concentration of bacteria solution and absorbance value was obtained by serial dilution plate counting method.

### Cell culture

The oral tongue squamous cell carcinoma cells SCC15 (supported by Chongqing Key Laboratory of Oral Diseases and Biomedical Sciences) were cultured in DMEM/F12 with 10% fetal bovine serum (Gibco, USA) containing 100U/ml penicillin and 100 μg/ml streptomycin at 37 °C and 5% CO_2_.

### Co-culture time and concentration

The *S. anginosus* supernatant in stable phases was used for the experiment, treating with centrifugation at 4000 rpm/min for 20 min and filtering twice with 0.22 μm filters. Then, the treated supernatant was called filtrate S and the bacterial medium THB was treated in the same manner as a control, called filtrate T. The Subsequent experiments were carried out with filtrate S and filtrate T. A series of concentrations of filtrate S were applied to SCC15, then an optimal co-culture time and concentration were obtained by CCK-8 assay.

### Experimental groups and manipulating approaches

Methods to set up SCC15/ *S. anginosus* (SCC15/S) experimental group, SCC15/THB (SCC15/T) conditional control group, SCC15/S+3-MA conditional control group, SCC15/3-MA negative control group and SCC15 blank control group. Meanwhile, SCC15 group did not do any treatment, while SCC15/S, SCC15/T and SCC15/3-MA acted with filtrate S, filtrate T and 3-Methyladenine (3-MA) for 16 h respectively; SCC15/S+3-MA pretreated with 3-MA for 4 h and then infected with filtrate S for 16 h.

### Autophagic response measurement

#### Monodansylcadaverine staining

Monodansylcadaverine (MDC) staining was an independent method to evaluate autophagy. SCC15 cells were seeded in confocal dishes. After each group was treated accordingly,10 μL MDC stain was added and mixed, and incubated at 37 °C for 20 min away from light; Then washed with 300μL 1X wash buffer twice. Pictures were obtained with the confocal microscope (Thermo, USA).

#### Real time PCR analysis

Total RNA was isolated from SCC15 cells using Trizol method (Beyotime, China). cDNA was synthesized by qScript cDNA synthesis kit (Sigma, USA) and primers synthesized by Wuhan Sevier Biotechnology Co., Ltd. The sequences are shown in Table 1. Quantitative gene expression was performed for Beclin1, LC3 using one-step RT-PCR kit (Takara, Japan). Expression values were normalized to GAPDH.

**Table 1:** Primers used in RT‐PCR analysis

Gene	Sequence (5′–3′)	Length (bp)
LC3	Sense AGCGAGTTGGTCAAGATCATCC	136
	Antisense CCTCGTCTTTCTCCTGCTCGTA	
Beclin1	Sense GAGCCATTTATTGAAACTCCTCG	162
	Antisense CCCAGTGACCTTCAGTCTTCG	
GAPDH	Sense ACTTTGGTATCGTGGAAGGACTCAT	255
	Antisense GTTTTTCTAGACGGCAGGTCAGG	

RT‐PCR, reverse‐transcription polymerase chain reaction

#### Western blot analysis

The cell samples were collected and lysed in RIPA with PMSF (Beyotime, China). Then, protein concentration was mearsured by BCA protein kit (Beyotime, China). The cell lysates were separated by 10% SDS–PAGE and transferred to the 0.22 μm PVDF membranes. The bands were detected by enhanced chemiluminescence (ECL) after the primary antibody at 4 °C for a night and second antibody at room temperature for 1 h. The antibodies used in this study include: anti-LC3, anti-Beclin1 (Cell Signaling, USA) and GAPDH (Beyotime, China).

### Cell proliferation analysis

Cells were seeded in a 96-well plate at a density of 5 × 10^3^/well and incubated at 37 °C for 24 h, then each group was treated accordingly. After that,10 μL CCK-8/well with 100 μL DMEM/F12 was added and incubated at 37 °C for 4 h. Absorbance was determined at 450 nm with a microplate reader.

### Cell cycle and apoptosis analysis

The cells were digested with trypsin and washed with cold PBS, then fixed overnight in 70% ethanol at − 4 °C. Ethanol-fixed cells were collected and washed with PBS, then cell counting. 10^6^cells of each group were centrifuged and 0.5 mL PI/RNase stain was added to cell pellets. After 30 min at room temperature in the dark, the cell cycle was analyzed with flow cytometre with a 488 nm argon laser.

Cell culture medium of each group was collected respectively and Cells were trypsinized with EDTA-free trypsin. Then washed cells with cold PBS twice and cell counting. 10^6^cells of each group were centrifuged and 400 μL 1 × AnnexinV binding buffer was added to resuspend cells. Afterwards, added  5 μL FITC and 10 μL PI per group for incubation at − 4 °C in the dark for 15 min and 5 min respectively.

### Cell migration and invasion assay

The cell migration was measured by wound healing assay. Scratched a line with pipette tip in the middle of each well and washed with PBS 3 times slightly. Then 2 mL of serum-free medium was added to each well and incubation at 37° C for 24 h. The pictures of each group were recorded at 0 h, 6 h, and 12 h under microscopy at 40 magnifications.

The cell invasion was measured by the transwell system (BD, USA). Treated cells were inoculated in the upper chamber with free-FBS medium, and F12 culture medium containing 10% FBS was added to the lower chamber. Additionally, an insert covered with Matrigel was used for invasion measurements. After 24 h, cells migrated to the opposite side of the insert were stained with DAPI and quantified.

### Statistical analysis

Data analysis was performed by SPSS 25.0 software. All values are calculated and expressed as mean ± standard deviation (SD). Anaylsis of Variance (ANOVA) and SNK-q test were used to compare between the groups. *P*-value < 0.05 was considered statistically significant.

## Results

### *S. anginosus* growth curve and concentration standard curve were detected and co-culture time and concentration were determined

The growth of *S. anginosus* is consistent with the general rule which contains four phases, including: 0–4 h lag phase, 4–16 h log phase, 16–24 h stable phase, and 24 h later as the decay phase (Additional file 1: Fig. S1). The stable phase 20 h bacterial solution was selected for experiment because the amount of primary and secondary metabolites of bacteria will reach the maximum in this period. Then, according to the concentration standard curve (Additional file 2: Fig. S2), the relationship between the concentration and the absorbance value can be obtained. The bacterial solution at 20 h was adjusted to the concentration corresponding to the growth curve to ensure the same concentration in each test. Furthermore, the results of CCK-8 assay showed that when a 1:1 relationship between filtrate S and cell culture medium (Additional file 3: Fig. S3) and co-cultured time was 16 h (Additional file 4: Fig. S4), the effect of inhibition to cells was the most obvious. Therefore, this concentration and co-cultured time were used for subsequent experiments.

### *S. anginosus* up-regulated autophagy activity of SCC15

**Fig. 1:**
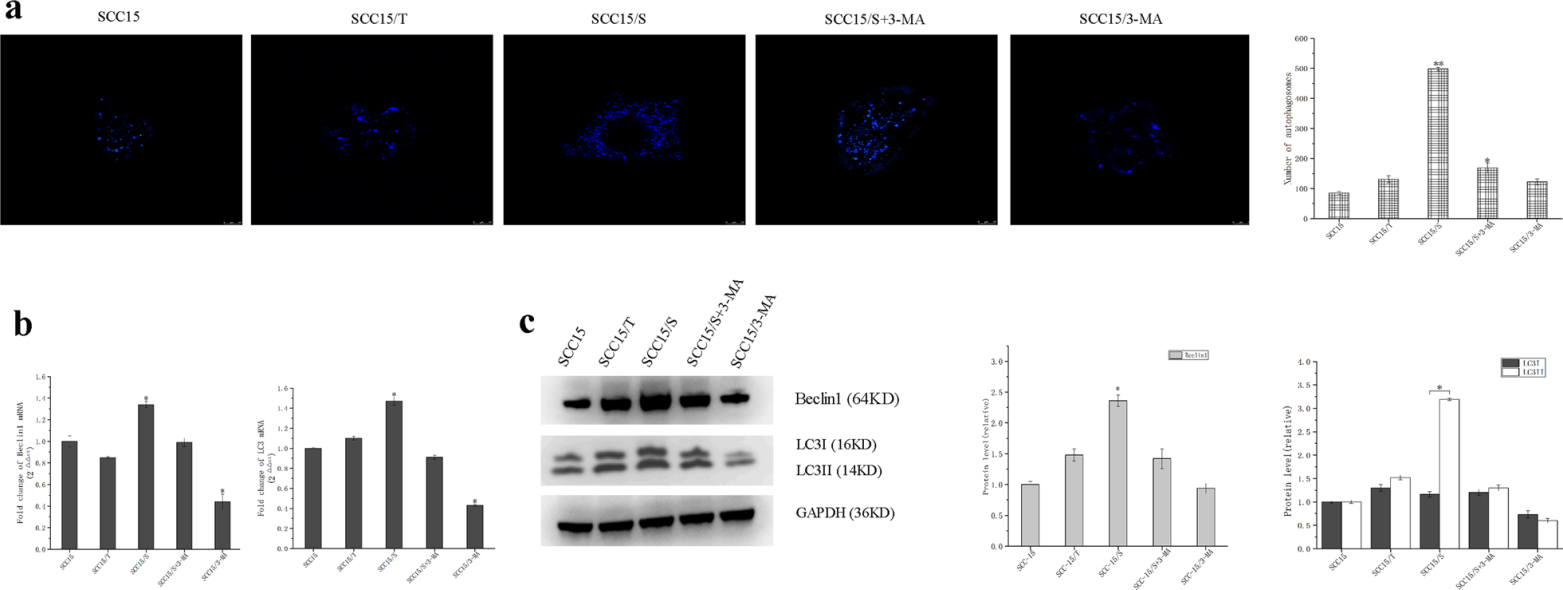
*S. anginosus* up-regulated autophagy activity of SCC15. **a** Autophagosomes in cells assessed using MDC staining (× 800 magnification). **b** The mRNA levels of Beclin1 and LC3 were assessed by qRT-PCR. **c** The autophagy-associated proteins levels of Beclin1, LC3I and LC3II were assessed using Western blot analysis, and normalized by GAPDH. Data were representative of three independent experiments and shown as mean ± SD, *: *P* < 0.05, **: *P* < 0.01 as versus blank control group

Autophagy is a physiological compensation process to maintain the homeostasis of eukaryotic cells. It can not only exert tumor suppressive effects, but also help tumor cells escape the body's metabolism. A recent study by Chen et al. has supposed that autophagy may inhibited the proliferation of cells [16]. To investigate whether autophagy participates in the action of *S. anginosus* on SCC15 cells, we first established a model system as the negative group by blocking the autophagy pathway with 3-MA, in which group autophagy activity was inhibited obviously. Vital staining was performed with MDC dyes, which a specific fluorescent marker for autophagic vacuoles showed that compared with SCC15 group, the number of autophagosomes was the highest in SCC15/S group, SCC15/S+3-MA group and SCC15/T group were decreased in turn, and SCC15/3-MA group was the least (Fig. 1a). What’s more, the amount of LC3I protein conversion to LC3II protein has also proved to be well correlated with the degree of autophagy [17]. We found that the mRNA expression of Beclin1 and LC3 were efficiently higher in SCC15/S group than other groups (*P* < 0.05) and significantly decreased in SCC15/3-MA (*P* < 0.05) (Fig. 1b). Meanwhile, the expression of autophagy-related protein Beclin1 was significantly enhanced in SCC15/S group and protein LC3 type II increased, type I decreased, showing the transformation from type II to type I. while these two proteins Beclin1 and LC3II were both decreased in SCC15/3-MA group (Fig. 1c; Additional file 5: Fig. S5).

### *S. anginosus* inhibits the proliferation and promotes apoptosis of SCC15

**Fig. 2:**
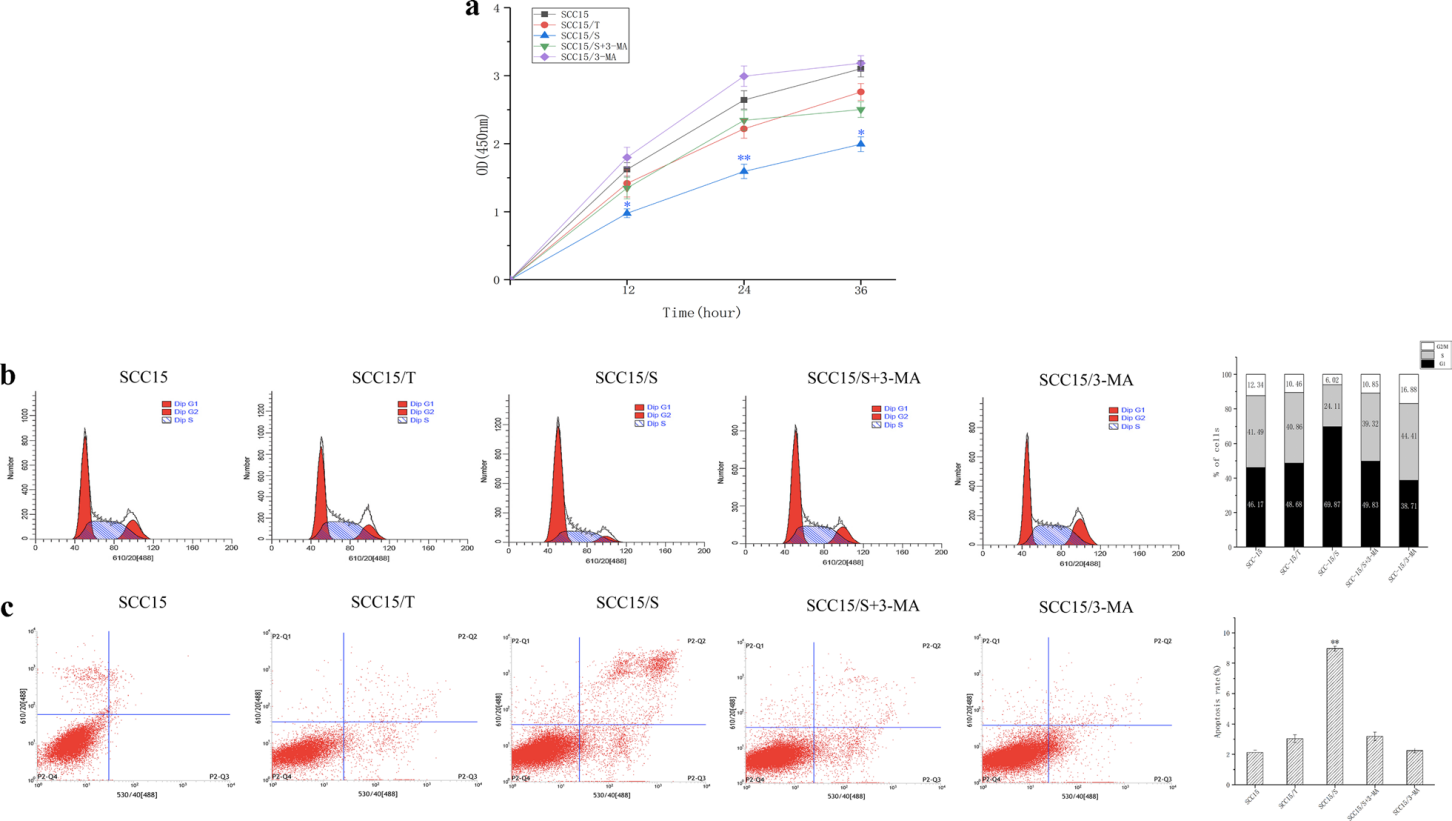
*S. anginosus* promotes apoptosis of SCC15 and inhibits the proliferation through a cell cycle arrest at the G1/S transition. **a** CCK-8 assay was performed to test cell proliferation of SCC15 cells. **b** The cell cycle distributions of SCC15 cells were analyzed with flow cytometry. **c** Cell apoptosis rate was measured by flow cytometry. Data were representative of three independent experiments and shown as mean ± SD, *: *P* < 0.05, **: *P* < 0.01 as versus blank control group

We evaluated the proliferative activity of SCC15 cells from 0 to 36 h by CCK-8 assay. The results indicated that the SCC15 cells exhibited reduced proliferation after incubation with filtrate S while SCC15/3-MA group revealed slightly increased (*P* < 0.05). Meanwhile, there was no significant difference between SCC15/T group and SCC15/S+3-MA group compared with the blank control group (Fig. 2a). When flow cytometry was used to analyze cell cycle, the results showed that *S. anginosus* abundant accumulation of SCC15 cells in the G1 phase (Fig. 2b). In addition, the number of apoptotic cells in SCC15/S group was significantly higher than that in the four control groups (Fig. 2c), and the difference was significant (*P* < 0.05).

### S. anginosus was involved in autophagy-mediated inhibition of migration and invasion

**Fig. 3:**
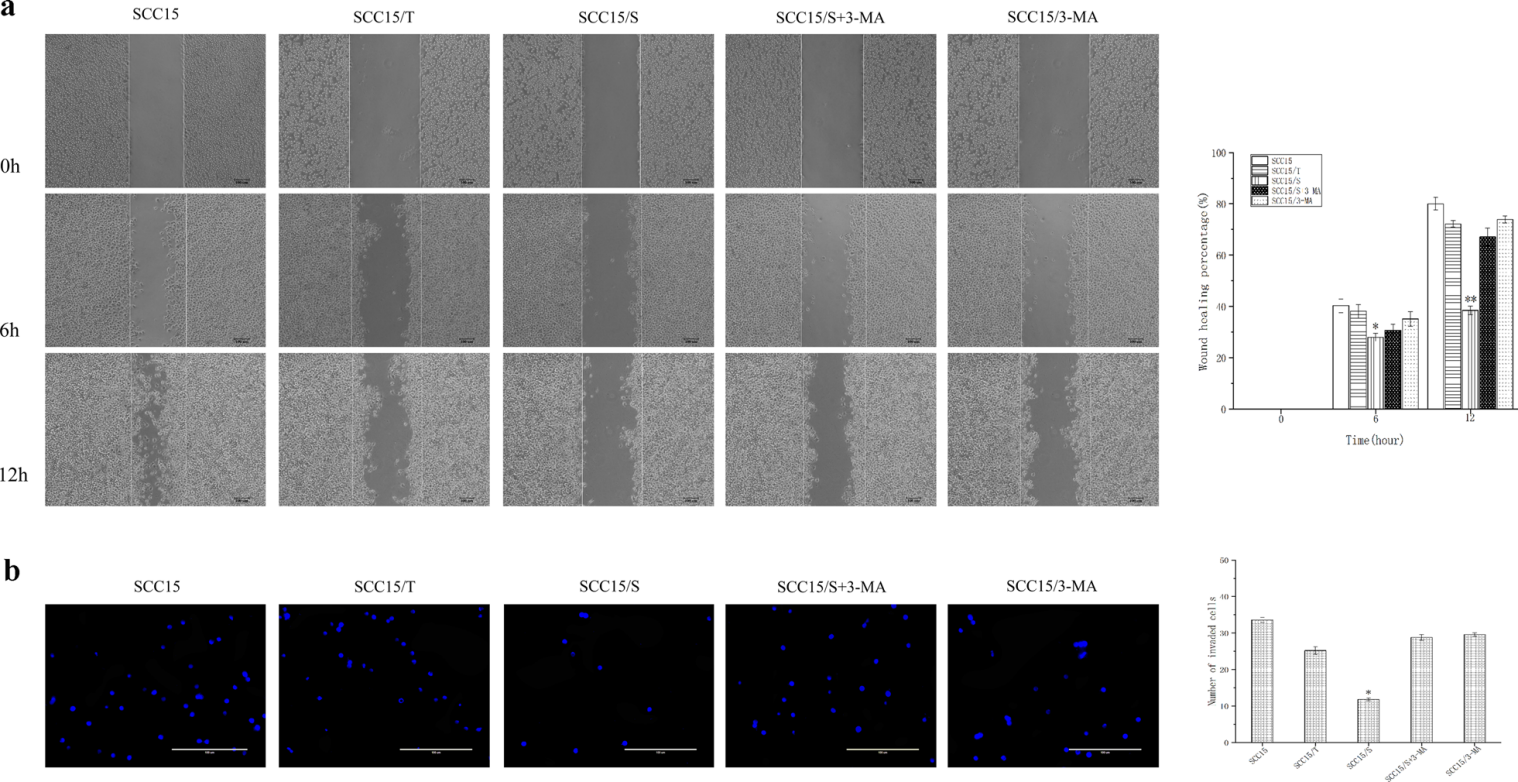
*S. anginosus* was involved in autophagy-mediated inhibition of migration and invasion. **a** Migratory ability of SCC15 cells was detected by wound healing assay. presence of autophagy in SCC15/S decreased migratory ability compared with control groups (*P* < 0.05). **b** Transwell migration assays manifested that up-regulated autophagy decreased numbers of migrated cells. Data were representative of three independent experiments and shown as mean ± SD, *: *P* < 0.05, **: *P* < 0.01 as versus blank control group

The results of wound healing assay showed that the healing area of SCC15/S group was lower than that of all control groups (*P* < 0.05, Fig. 3a), indicating that the mobility of SCC15/S group was decrease significantly and highly suggesting that *S. anginosus* was a key factor in the decline of SCC15 cell motility.

To further evaluate the function of metabolic products of *S. anginosus* on SCC15 cells invasive ability, transwell chambers coated with matrigel were used. We found that filtrate S of *S. anginosus* resulted in a significant decrease in invasivity compared with control groups (*P* < 0.05). However, opposite result was dramatically observed when autophagy was inhibited (*P* < 0.05, Fig. 3b).

## Discussion

The large number of microbiome in the body affects the susceptibility to cancer, partly because its metabolites or co-metabolites have important effects on the function of immune cells. Studies report that 15 to 20 percent of cancer cases are associated with microbial infections [18], but the role of microorganisms in tumor genesis and development is still controversial today. There were some highlighted questions about whether oral microbiome changes are an important risk factor for oral cancer development [19]. According to the results, patients with oral leukoplakia (OLK), a lesion with malignant potential, were more enriched with *Fusobacteria* compared to normal tissue from the same patients [20]. In addition, the opinion that microbiome changes preceded the malignant transformation process was confirmed, supporting the possible role of microbiome changes in the pathogenesis of disease [9].

*Porphyromonas gingivalis* is one of the most studied oral microorganisms in vivo and in vitro. Studies have found that continuous stimulation of normal epithelial cells by this kind of bacteria can lead to tumour-like changes in cells [21]. However, the reverse effect was reported in other study [22]. Some researchers have summarized the role of oral microbiota in cancer development. The results suggested that among *streptococci, S. anginosus* seems to be an especially relevant marker of head, neck, and esophageal cancers and more common in oral squamous cell carcinoma. They also pointed out three mechanisms of oral microbiota in cancer pathogenesis. The first is bacterial stimulation of chronic inflammation. The second is activation of NF-κB and inhibition of cellular apoptosis; And the third is the carcinogens produced by bacteria [23].

*S. anginosus*, as a human symbiotic bacterium, was first proposed in 1983 that it may be involved in the occurrence and development of oral infectious diseases [14]. In recent years, many relevant studies have claimed that the detection rate of this bacterium in OSCC tissues has increased [24], but its actual effect and mechanism on tumor cells have not been reported. SASAKI et al. isolated and purified a new bioactive antigen SAA (*S. anginosus* antigen) from *S. anginosus* supernatant. They found that SAA stimulated macrophages to produce high concentrations of nitric oxide (NO) and various inflammatory factors. NO interacted with O^2^ or O^2^- to form reactive nitrogen species (RNS), which leads to a DNA damage response by DNA base oxidation and nitration. In addition, the β-hemolysin of *S. anginosus* and the streptolysin S (SLS) of Streptococcus pyogenes are homologous series encoded by a similar gene cluster; SLS is a highly toxic cytolysin, which help bacteria to cross the epithelial barrier with a tissue damage and also can against the immune clearance of hosts [25]. Based on the reported toxic effects of metabolites of this bacteria, we selected *S. anginosus* supernatant of stable period for treatment and retained the metabolites in the supernatant for related experiments.

Autophagy is a physiological compensation process which cell removes damaged proteins and organelles to maintain its homeostasis [26]. Stress reactions such as starvation, hypoxia and microbial infection can stimulate autophagy. Many literatures have stated the relationship between tumor, microorganism and autophagy [27–29]. However, it is not clear whether the decrease in autophagy activity observed in malignant cells is mechanically significant or merely incidental to the progression of malignancy. Invasion and metastasis are the critical markers in the development of cancer, and active cell migration plays an essential role in the invasion and metastasis cascade of cancers. Our study confirmed that *S. anginosus* reduced the proliferation, migration and invasion of SCC15 cells and promoted cell apoptosis; Furthermore, it has been found that Beclin1 located in the cytoplasmic endoplasmic reticulum is involved in the formation of autophagic vesicles and also an important factor in inducing autophagic death of tumor cells. Structurally, Beclin1 has the BH3 region that constitutes the apoptotic bcl-2 protein family, and it is a specific receptor of apoptosis, so it plays an important role in promoting apoptosis [30]. In this study, MDC staining, qPCR and western blot results also confirmed increased autophagy activity and up-regulated expression of Beclin1 during this process, which was consistent with our results of increased apoptosis rate.

Picardo SL et al. suggested that microbial regulation may affect the course of the disease [31]. In patients with advanced cancer treated with immunotherapy, resistance is associated with microbiome abnormalities and antibiotic treatment. There is certainly evidence that the gut flora plays a key role in the response of cancer patients to chemoradiotherapy and immunotherapy. Microbiota transplantation (MT), including fecal microbiota transplantation (FMT) and selective microbiota transplantation (SMT), may improve the effect of anti-cancer treatment and/or reduce the related side effects [32]. Viaud et al. demonstrated that cyclophosphamide causes discontinuity of the intestinal barrier and subsequently promotes selective transfer of specific Gram-positive bacteria to secondary lymphoid organs. These transplanted bacteria can enhance the anti-cancer adaptive immune response of T cells [33].

A new study indicates that the composition of the patient's intestinal flora is an important factor in regulating the host's response to anti-PD-1 / PD-L1 or anti-CTLA-4 immunotherapy [34]. In addition, the view that regulation of the microbiome can be used in cancer treatment was proposed [35]. The study in a mouse model of colonic carcinogenesis showed that oral intake of probiotics containing lactobacilli can reduce IL-17-producing T cells and inhibit proliferation and tumor formation, which may be achieved by changing the gut microbiome. National Institute of Health reconstructed the laboratory mice with natural “wild-type” microbiota, and increased resistance to mutagen and inflammation-induced colorectal tumorigenesis were found [36]. Wang et al. observed that *S. anginosus* stimulated peripheral blood of OSCC patients and healthy people, and found that CD8 T cells were significantly higher in OSCC patients, proving that Streptococcus-reactive CD8 T cell responses might contribute to antitumor immunity in OSCC patients [37].

Above all, microbiome profoundly affects immune development and carcinogenesis. microbiome-modulating agents are poised to become bona fide anti-cancer strategies: immunotherapy. Immunotherapy derives from the recognition of the synergy between host and microbe. In 1850, several German physicians found that some cancer patients with active infections showed signs of tumor regression. In 1900, Coley test bacterial extracted on patients with bone cancer which was one of the first immunotherapies [38]. For the past three decades, several bacteria-based cancer treatments have emerged, and bacterial vaccines expressing tumor antigens have been shown to be effective in preclinical studies. Bacillus Calmette-Guerin (BCG) was used in the treatment of non-muscular invasive bladder cancer, where directly transmitted live bacteria enter the bladder, causing inflammation and triggering an anti-tumor immune response [39]. Synthetic biology approaches to cancer care hold enormous potential, especially those that make use of bacteria. These methods involve reengineering of bacterial cells for the delivery biomolecules in host reactions. The concept that microorganisms can invade cancer cells to target and disrupt critical cancer pathways has been demonstrated. The next step will be to use robust preclinical models for evaluation.

Oral microflora is composed of many microorganisms, among which there are complex interrelationships. In our study, the outcome of the effect of other microorganisms on the interaction of *S. anginosus* and the exact clinical role of *S. anginosus* are not clear. Our experiment is only an initial exploratory experiment. So, the interaction of *S. anginosus* with more OSCC cell line and normal cells and vivo research should be verified by further experiments.

## Conclusions

Our study showed the inhibitory effect of *S. anginosus* on cancer cells. We believe that the results of such studies, including the present ones, could provide an entry point for future studies of cellular interactions between *bacteria* and cancer. In addition, future studies should be designed to elucidate the impacts of *S. anginosus* on the chemotherapeutic responses of oral cancer cells, of which outcomes will have a significant impact on the clinical treatment of oral cancer.

## Supplementary Information

**Additional file 1.** Growth curve of Streptococcus anginosus. The growth cycle of S.anginosus including: 0–4 h lag phase, 4–16 h log phase, 16–24 h stable phase, and 24 h later as the decay phase. The stable phase 20 h bacterial solution was selected for the next experiments because of the maximum metabolites in this period.

**Additional file 2.** Standard curve of Streptococcus anginosus concentration. The relationship between concentration and absorbance value can be obtained according to the formula. And the absorbance value of stable phase 20 h bacterial solution was a standard in each experiment.

**Additional file 3.** Determination of the co-culture concentration. A series of concentrations of filtrate S were applied to SCC15, and the results of CCK-8 assay showed that when a 1:1 relationship between filtrate S and cell culture medium, the most significant inhibition was obtained.

**Additional file 4.** Determination of the co-culture time. According to the growth curve, the optimal ration of filtrate S and cell culture medium was applied to SCC15, and the cell viability measured at the corresponding time point of the growth curve. Co-culture for 16 hours had the most obvious inhibitory effect on cells.

**Additional file 5.** Original blot images.The autophagy-associated proteins levels of Beclin1, LC3I and LC3II were assessed using Western blot analysis, and normalized by GAPDH.

## Data Availability

The datasets used during the current study are available from the corresponding author on reasonable request.

## Abbreviations

*S. anginosus*: Streptococcus anginosus
OSCC: Oral squamous cell carcinoma
3-MA: 3-Methyladenine
MDC: Monodansylcadaverine
ANOVA: Anaylsis of Variance
OLK: Oral leukoplakia
SAA: Streptococcus anginosus antigen
NO: NItric oxide
RNS: Reactive nitrogen species
SLS: Streptolysin S
MT: Microbiota transplantation
FMT: Fecal microbiota transplantation
SMT: Selective microbiota transplantation
