# Supplementary figures and images for: Effect of Streptococcus anginosus on biological response of tongue squamous cell carcinoma cells
Source: BMC Oral Health. 2021 Mar 20;21:141. doi: 10.1186/s12903-021-01505-3 (PMC7981962; doi:10.1186/s12903-021-01505-3)

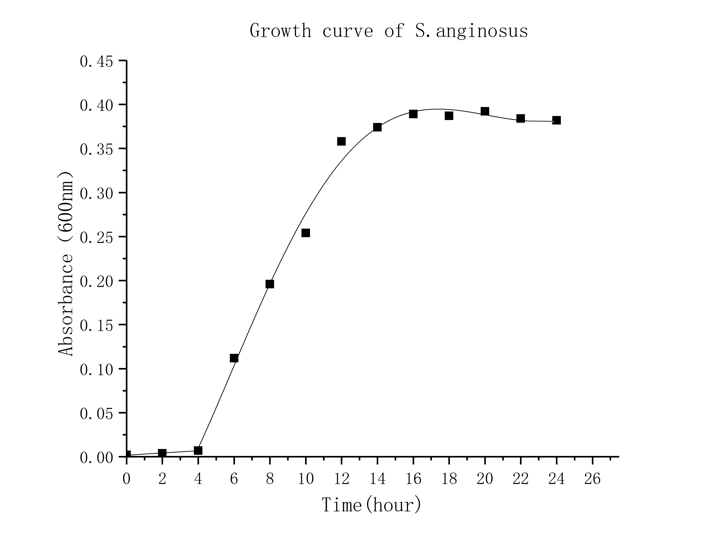

Supplement: Supplementary file 1 — Additional file 1. Growth curve of Streptococcus anginosus. The growth cycle of S.anginosus including: 0–4 h lag phase, 4–16 h log phase, 16–24 h stable phase, and 24 h later as the decay phase. The stable phase 20 h bacterial solution was selected for the next experiments because of the maximum metabolites in this period. [file 12903_2021_1505_MOESM1_ESM.tif]

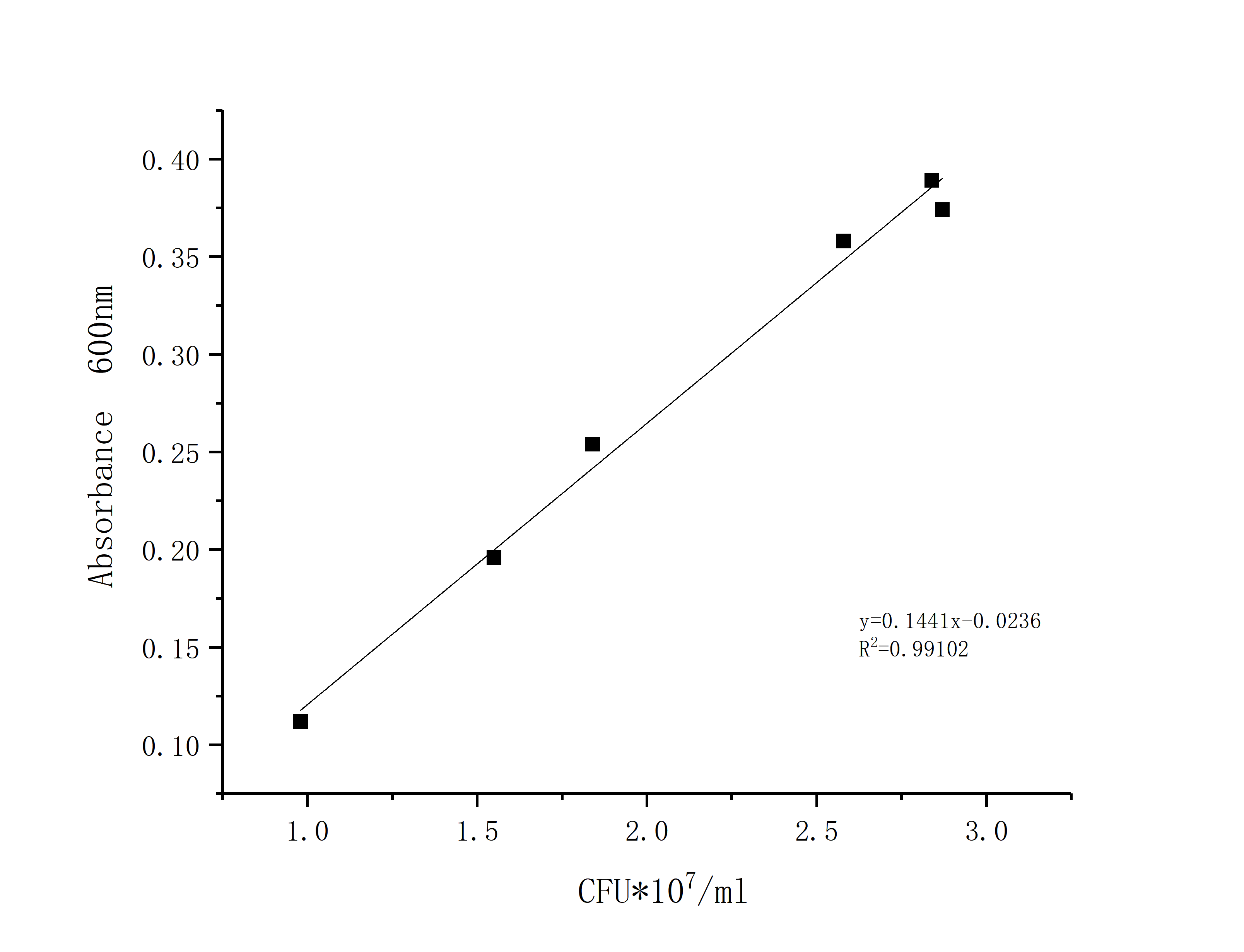

Supplement: Supplementary file 2 — Additional file 2. Standard curve of Streptococcus anginosus concentration. The relationship between concentration and absorbance value can be obtained according to the formula. And the absorbance value of stable phase 20 h bacterial solution was a standard in each experiment. [file 12903_2021_1505_MOESM2_ESM.tif]
