# Supplementary material for: Effect of Streptococcus anginosus on biological response of tongue squamous cell carcinoma cells
Source: BMC Oral Health. 2021 Mar 20;21:141. doi: 10.1186/s12903-021-01505-3 (PMC7981962; doi:10.1186/s12903-021-01505-3)
Supplement: Supplementary file 5 — Additional file 5. Original blot images.The autophagy-associated proteins levels of Beclin1, LC3I and LC3II were assessed using Western blot analysis, and normalized by GAPDH. [file 12903_2021_1505_MOESM5_ESM.docx]

64**-**

KDa

**SCC15**

**SCC15/T**

**SCC15/3-MA**

**SCC15/S+3-MA**

**SCC15 /S**

1. Beclin1

**SCC15/S+3-MA**

**SCC15/3-MA**

**SCC15/T**

**SCC15**

**SCC15 /S**

KDa


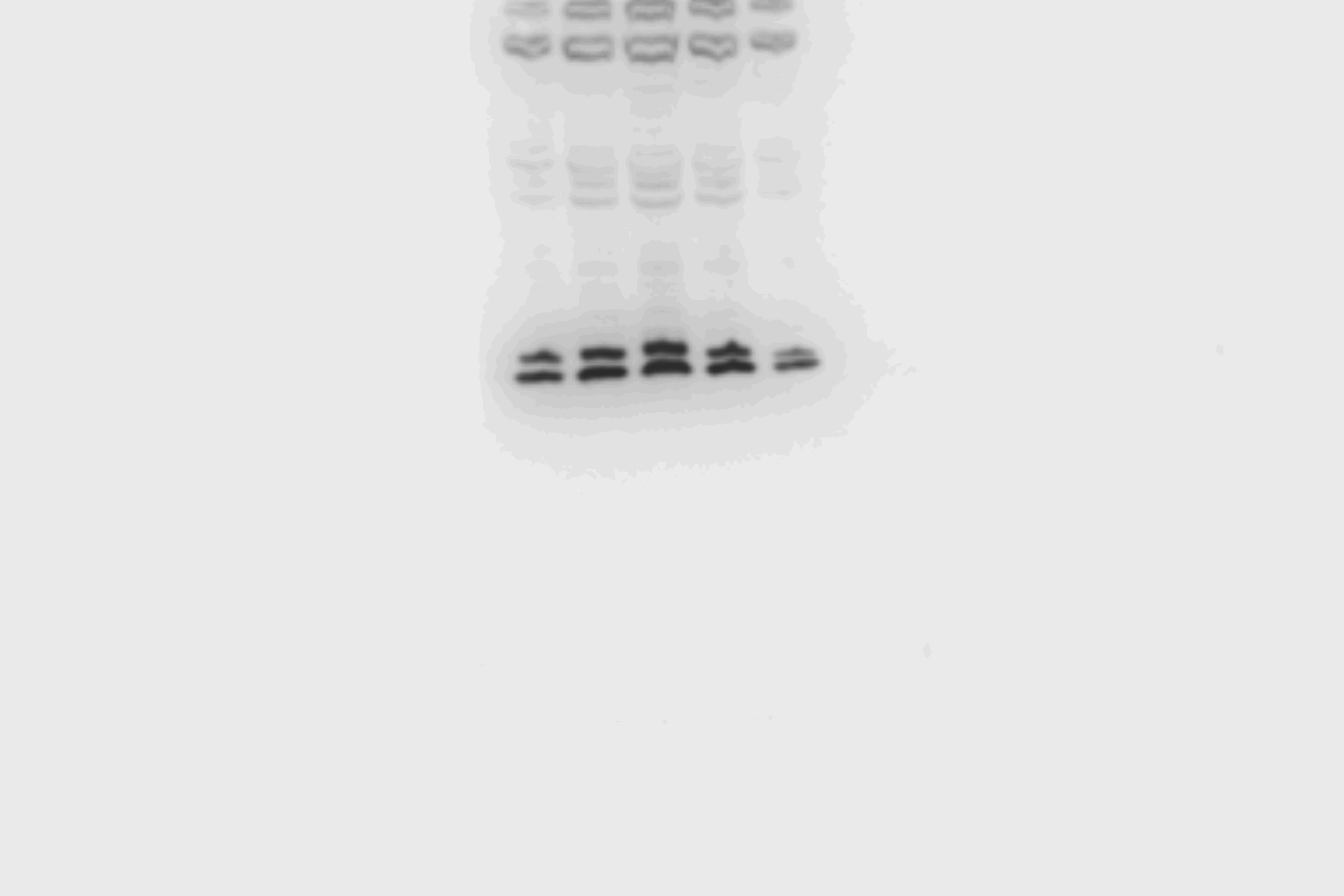


14**-**

16**-**

1. LC3I&LC3II

**SCC15/3-MA**

**SCC15/S+3-MA**

**SCC15 /S**

**SCC15/T**

**SCC15**





36**-**

KDa

1. GAPDH
